# Depolymerizable Elastomeric Polyolefin Thermosets with Great Extensibility

**DOI:** 10.1021/acsmaterialslett.5c01249

**Published:** 2025-12-08

**Authors:** Gadi Slor, Quy Ong Khac, Laura Roset Julià, Youwei Ma, Francesco Stellacci

**Affiliations:** † Institute of Materials, 27218 École Polytechnique Fédérale de Lausanne (EPFL), Lausanne 1015, Switzerland; ‡ Institute of Bioengineering, École Polytechnique Fédérale de Lausanne (EPFL) Lausanne 1015, Switzerland

## Abstract

The development of high-performance rubber materials has been a long-standing pursuit; currently, this has to go hand-in-hand with the design of polymers that are in some way recyclable. In this work, we report a class of thermosetting polyolefin elastomers synthesized via ring-opening metathesis polymerization of cycloheptene cross-linked with dicyclopentadiene. These cross-linked thermosets exhibit markedly enhanced chemical resistance, mechanical robustness, thermomechanical stability, and elasticity compared to those of their linear analogue. Notably, they demonstrate extraordinary extensibility, with strain at break exceeding 1700%, attributed to strain-induced crystallization confirmed by small- and wide-angle X-ray scattering analyses. Moreover, the elastomers are depolymerizable in the presence of Grubbs Catalyst second Generation, enabling recovery of cycloheptene in good yields of 77%–92%. Lastly, we show that the (thermo)­mechanical properties of the materials could be further enhanced through the incorporation of activated charcoal, and the resulting composites still retain a certain level of depolymerizability, affording cycloheptene in a yield of 60%.

## Graphical abstract

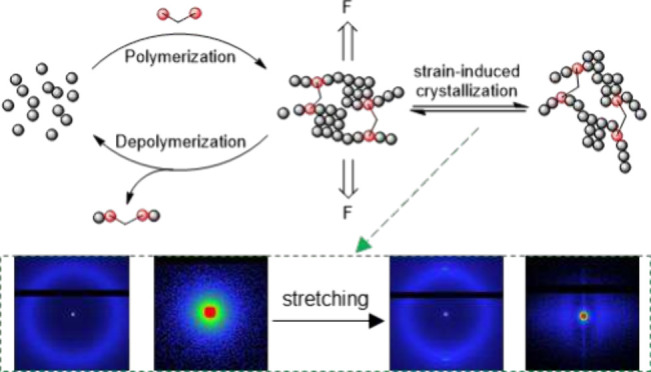

Since Hofmann et al. successfully polymerized isoprene in 1909, synthetic rubbers have played indispensable roles across a wide range of applications on account of their excellent elasticity. In 2023 alone, global production reached 14 million metric tons, representing a 100% increase over the past two decades.^1^ In contrast to rapid growth, the end-of-life management of synthetic rubbers remains insufficiently addressed, leading to substantial resource loss and environmental concerns. For example, tires, accounting for 60%–70% of the rubbers consumed,^2^ are discarded in vast quantities each year, and estimated to reach 1.2 billion tons by the 2030s.^3^ 17% of the scraped tires end up in landfills, and a third of them is subjected to pyrolysis—a chemical recycling process involving heating the rubbers at high temperatures. Although the pyrolysis process leads to the recovery of olefinic gases and oils, it requires high energy input and releases hazardous byproducts, such as benzene, dioxins, and furans.^4^

Recent advancements in ring-opening metathesis polymerization (ROMP) of cyclic olefins provide a promising solution to the above issue, as it produces elastomeric polyolefins that can undergo ring-closing depolymerization to recover the starting materials after their service life.^5^ So far, many efforts have been dedicated to addressing the stability–depolymerizability tradeoff,^6–8^ the synthesis of novel monomers and catalysts,^9–11^ and engineering functionalities to polymers.^9,12–14^ Reported cyclic olefins are primarily in five-,^15,16^ six-,^17^ eight-membered,^7,8,18^ or even larger ring sizes,^11,19^ with very recent works from the Sun lab also showing the feasibility of seven-membered cycloheptene^20^ and its derivatives^6^ in the synthesis of depolymerizable polyolefins. These investigations have laid a solid foundation in accessing sustainable, chemically recyclable rubbers, potentially contributing to a circular materials economy. However, a critical limitation persists: the majority of reported works yield linear and/or branched polymer architectures,^5–20^ —which deviate from the real-world application scenarios, where rubbers are typically cross-linked and often reinforced with composite fillers such as carbon black or silica to meet performance requirements.^21–24^

Previous reports have highlighted that the introduction of cross-linking structure could improve various properties (e.g., chemical resistance, mechanical properties, elasticity) of the linear polymers,^25–27^ with the mechanical properties further improved, or additional functionalities emerged through the incorporation of composite fillers.^28–30^ Based on these insights, we hypothesize that engineering cross-links and composite fillers into ROMP-derived rubbers could yield materials with superior performance characteristics. However, the impact of their joint use on the depolymerizability of the materials remains largely unexplored. Such investigations may provide a technical assessment on the viability of this type of materials as sustainable alternatives to conventional, nonrecyclable rubbers.

In this work, we designed rubbers with cross-linked architecture based on a ROMP-derived polyolefin, known as polyheptenamer (**PHP-x**), whose depolymerizability has been demonstrated recently by Sun and co-workers.^6,20^ These materials are synthesized via copolymerization of cycloheptene (**CH**) and a difunctional cross-linker dicyclopentadiene (**DCPD**) (Scheme 1, left), and parameter **x** represents the molar feed ratio of **DCPD** to **CH**. This is followed by systematic investigations into the impact of cross-link density on various properties, of **PHP-x** including chemical resistance, thermal, mechanical, and thermomechanical properties, elasticity, as well as depolymerizability. Remarkably, the **PHP-x** films show great extensibility, with strain at break reaching above 2000%. Small- and wide-angle X-ray scattering (SAXS/WAXS) analyses reveal that this high extensibility arises from strain-induced crystallization (SIC) in the polymer networks (Scheme 1, right). Lastly, we showed that the (thermo)­mechanical properties of **PHP-x** are further improved through blending with activated charcoal (**AC**), and the resulting composite is still depolymerizable.

**Scheme 1:**
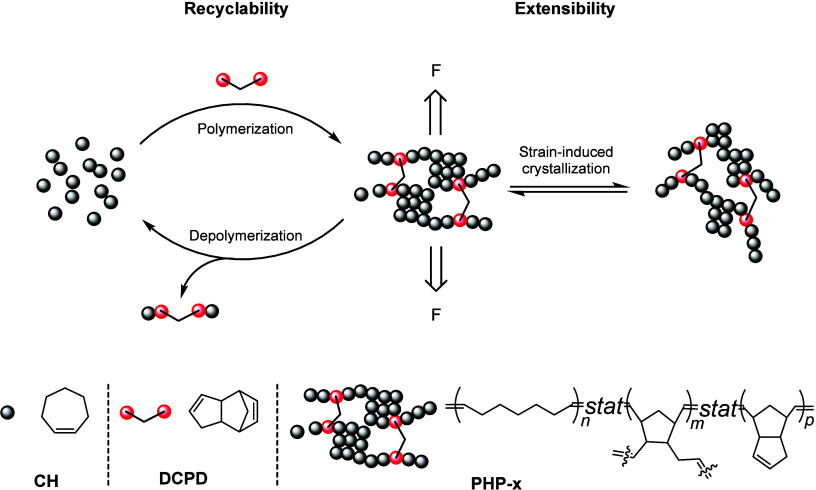
Copolymerization of **CH** and **DCPD** To Form **PHP-x** That Can Undergo Strain-Induced Crystallization upon Applying an External Force (F) and Also Can Depolymerize Back to Initial **CH**

The **PHP-x** films were synthesized in a two-step process adopted from the pioneering work by Johnson et al.^31^ First, **CH** and **x** mol % **DCPD** were polymerized in the presence of Grubbs Catalyst second Generation (G2; 0.03 mol %) and butylated hydroxytoluene (BHT; 1 wt%) at room temperature in an argon-filled glovebox, followed by post-curing in a drying oven at 120 °C for 30 min (Figure 1a). BHT was added to avoid nonspecific olefin cross-linking in the film during the thermal treatment. Among them, **PHP-0%** has no addition of **DCPD** cross-linker during the synthesis and should be in a linear polymer architecture. The chemical structure of **PHP-x** was confirmed by Fourier transform infrared (FTIR) analysis, showing characteristic C–H stretching and bending vibrations at 3000–2700 cm^–1^ and 1500–1400 cm^–1^, respectively (). Due to the chemical similarity between the cross-links and the main-chain linkages, the FTIR spectra of the various **PHP-x** samples almost overlap (), making it difficult to distinguish between linear and cross-linked polymers solely on the basis of spectral data. Hence, we performed a swelling experiment by immersing the films in their good solvent dichloromethane (DCM) for 24 h with solvent renewal every 12 h (). The results show that the **PHP-0%** sample dissolves completely, and **PHP-1%** undergoes significant dissolution, while **PHP-3%** and **PHP-5%** films exhibit substantial swelling but no significant dissolution (). This indicates the presence of cross-link architecture in the latter three polymers. Among the cross-linked polymers, the gel fraction increases significantly from 14% in **PHP-1%** to 43% in **PHP-3%**, and then to 84% in **PHP-5%** (see ), suggesting increased cross-link density. The chemical resistance of the cross-linked film was then evaluated by putting **PHP-3%** in various organic solvents including toluene, acetone, chloroform, acetonitrile, ethanol, and dimethyl sulfoxide (DMSO) for 24 h (). The film swelled over time but remained structurally intact in all cases, reflecting the robustness of the networks and its good resistance to these chemicals.

**Figure 1:**
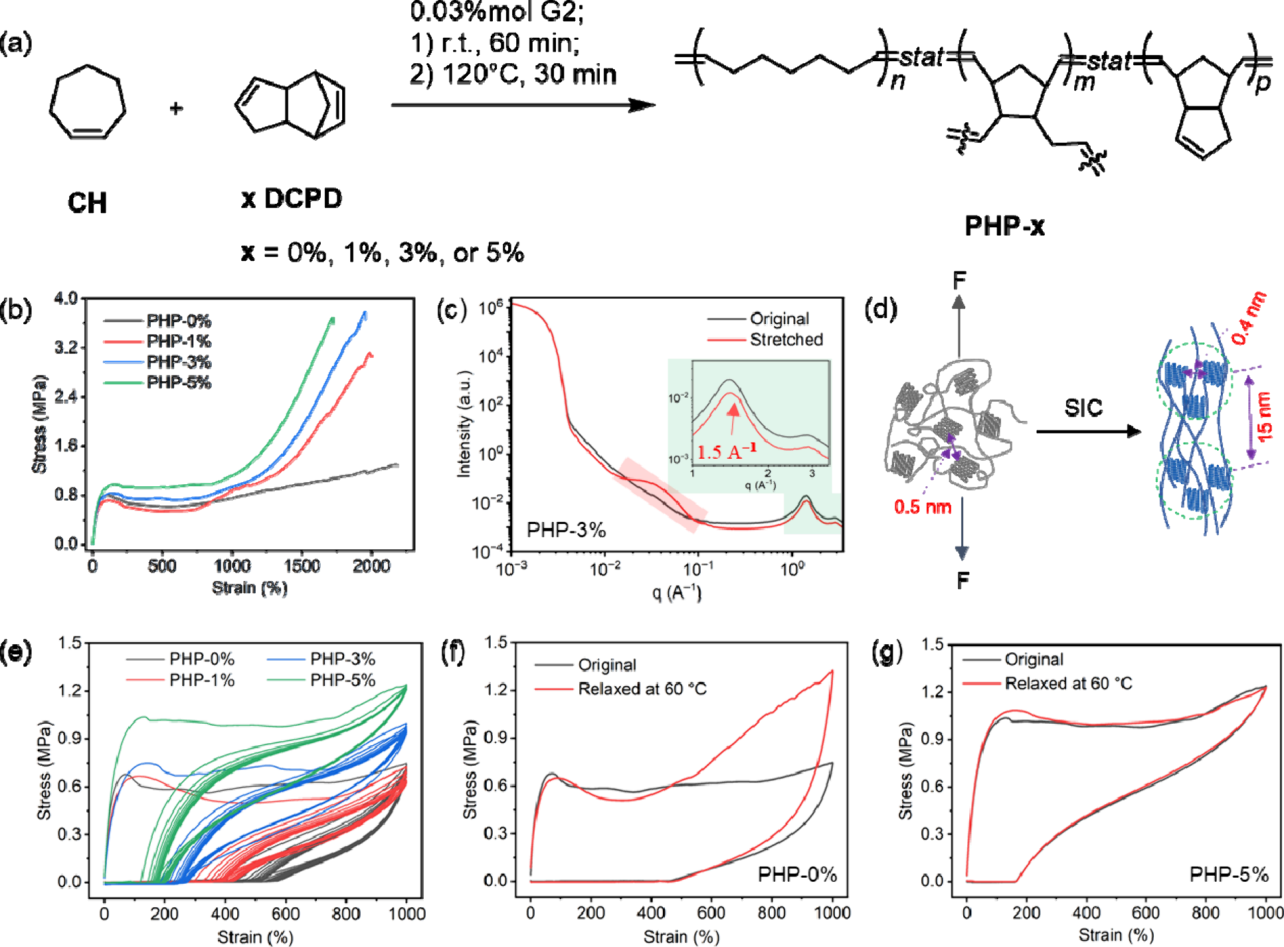
(a) Synthesis of **PHP-x** through ring-opening metathesis polymerization of **CH** and **x** molar equivalent of **DCPD** in the presence of Grubbs’ Second-Generation catalyst (G2). (b) Stress–strain curves of **PHP-x**. (c) 1D-SAXS/WAXS profiles of **PHP-3%** films before and after being stretched to breakage. (d) Schematic illustration of the structure variation of **PHP** during strain-induced crystallization (SIC). (e) Cyclic tensile curves of **PHP-x** films at a maximum loading strain of 1000% for 10 consecutive cycles. Cyclic tensile curves of the first cycle of (f) **PHP-0%** and (g) **PHP-5%** films before and after undergoing cyclic tensile testing at a maximum loading strain of 1000% for 10 consecutive cycles and then relaxation at 60 °C for 30 min.

The thermal properties of **PHP-x** were explored by thermogravimetric analysis (TGA) and differential scanning calorimetry (DSC). TGA curves reveal that all samples exhibit similar thermal decomposition behavior, with a 5% decomposition temperature exceeding 400 °C (), indicating excellent thermal stability. DSC traces show that all polymers display a distinct endothermic peak below 25 °C, corresponding to their melting temperature (*T*_m_), which indicates that the materials are in an amorphous state at room temperature (). Upon increasing the **DCPD** content (i.e., **x**), both the *T*_m_ and fusion enthalpy (Δ*H*_c_)—calculated by integrating the melting peak—gradually decrease, from 22.2 °C and 29.2 J g^–1^ of **PHP-0%** to 15.8 °C and 17.5 J g^–1^ of **PHP-5%**, respectively. This trend reveals that the introduction of cross-links diminishes the crystallization ability of the polymer networks, probably by restricting chain mobility, a finding that has been reported previously.^23,32,33^

We then investigated the thermomechanical properties of the **PHP-x** films by dynamic mechanical analysis (DMA). DMA traces of all samples exhibit a sharp decline in storage modulus (*E*′), accompanied by a peak in tan δ plots at the temperature of 33–55 °C (), that marks the melting of the crystalline domains in **PHP-x**. The *E*′ decline emerges at a lower temperature as the value of **x** increases, suggesting the decreased *T*_m_, a trend consistent with the DSC result (). However, the *T*_m_ values obtained by these two techniques differ by ca. 25 °C (), which is primarily attributed to the 1 Hz of oscillating force employed in the DMA measurement.^25^ Following the *E*′ transition, all samples exhibit a rubbery plateau. The plateau regime is steady for **PHP-3%** and **PHP-5%** as a function of temperature, while it is slanted for the other two samples (). It reflects the formation of robust cross-linked networks in **PHP-3%** and **PHP-5%**, while they are absent in **PHP-1%** and **PHP-0%** samples, which echoes the phenomenon in the swelling experiment: **PHP-3%** and **PHP-5%** maintain their network integrity in DCM, and the other two undergo complete or substantial dissolutions (). Within the rubbery regime, **PHP-3%** and **PHP-5%** films exhibit higher *E*′ values compared to **PHP-0%** and **PHP-1%** (), supporting their higher cross-link densities.

The mechanical properties of the **PHP-x** films were initially studied by uniaxial tensile testing (see Figure 1b, as well as ). The resulting stress–strain curves for all samples display typical features of semicrystalline polymers, encompassing an initial elastic region within 100% strain, followed by yielding deformation with modest or no strain hardening. The Young’s moduli of **PHP-x** range from 2.6 to 2.8 MPa, first see a decrease from 2.8 MPa of **PHP-0%** to 2.6 MPa of **PHP-1%**, which then increase to 2.8 MPa again for **PHP-3%** and **PHP-5%** (). A similar “decrease–increase” trend is observed in the yield stress: 0.8 MPa (**PHP-0%**) → 0.7 MPa (**PHP-1%**) → 0.9 MPa (**PHP-3%**) → 1.0 MPa (**PHP-5%**) (). The initial reduction in both Young’s modulus and yield stress from **PHP-0%** to **PHP-1%** can be attributed to the lower crystallinity of the latter (), whereas the subsequent increases for **PHP-3%** and **PHP-5%** are due to their higher cross-link densities (). Meanwhile, the stress at break increases substantially with cross-linking, rising from 1.2 MPa in **PHP-0%** to 4.2 MPa in **PHP-3%**—representing an over 3-fold improvement (). Evidently, the improvement is attributed to the presence of a strain-hardening regime in the cross-linked polyolefins (Figure 1b), which underscores the reinforcing role of cross-links. Further increasing the cross-link density to **PHP-5%** leads to a slight decline in stress at break (3.8 MPa), probably due to excessive network constraints that restrict polymer chain mobility.^22,34^ The restricted chain mobility is also reflected in the gradual decrease in strain at break (). When benchmarking our materials against other industrially relevant rubbers including natural rubber, styrene–butadiene rubber, ethylene propylene diene monomer, nitrile rubber, and polydimethylsiloxane (PDMS),^35–37^ we found that the **PHP-x** has comparable mechanical strength to PDMS while demonstrating superior strain at break (), indicating its potential as a viable PDMS alternative. Moreover, tensile rate-dependent measurements reveal that increasing the tensile rate from 100 to 500 mm min^–1^ for **PHP-3%** sample results in a higher stress at break but reduced strain at break ().

All **PHP-x** films demonstrate exceptional extensibility, with strain at break exceeding 1700% (see Figure 1b, as well as ). These values exceed those reported for most synthetic and natural rubbers (50%–1500%),^35,38–40^ which encourages us to elucidate the underlying mechanisms. To this end, simultaneous wide- and small-angle X-ray scattering (SAXS/WAXS) experiments were performed on the **PHP-3%** film before and after tensile testing (see Figure 1c, as well as ). The scattering profile of the unstretched film exhibits one sharp Bragg peak at q* = 1.38 Å^–1^, supporting the presence of crystal domains with a periodicity (*D*) of ca. 0.5 nm (calculated as *D* = 2π/*q* ^*^). Upon stretching, two additional shoulder peaks appear at *q* ^*^ = 1.5 and 0.04 Å^–1^ (Figure 1c). Concurrently, the 2D-SAXS pattern changes from circular to elliptical, and the 2D-WAXS pattern reveals the appearance of two arcs aligned with the stretching direction (). These observations suggest that the crystal domains exhibit a new *D* of 0.4 nm, and their distribution in the polymeric substrate turn from isotropic to anisotropic, showing a long *D* of 15 nm (Figure 1d). It further indicates that the **PHP-3%** film undergoes strain-induced crystallization, resulting in the formation and alignment of new crystal domains along the direction of deformation. These crystallites potentially serve as reinforcing elements within the polymer matrix, enhancing its mechanical integrity and contributing to the material’s remarkable stretchability—a key attribute of high-performance elastomers.^41–43^

To explore how the crystallites behave during reversible polymer deformation, simultaneous SAXS/WAXS measurements coupled with cyclic tensile testing were then conducted on the **PHP-3%** film.^44^ Upon increasing the tensile strain, the intensities of newly emerged Bragg peaks at *q* ^*^ = 1.5 and 0.04 Å^–1^ increase (), while the elliptical pattern in the 2D-SAXS, together with the arcs in the 2D-WAXS, intensifies (). These phenomena reflect more crystallites formed with an increasing strain. Subsequent retraction of the force on the **PHP-3%** gradually weakens both the elliptical SAXS pattern and the arcs in the WAXS, which respectively revert to an almost circular pattern and disappear when the external force was fully removed (). This is accompanied by a decrease in the intensities of the two Bragg peaks; Notably, the complete unloading leads to the disappearing of the peak at q* = 1.5 Å^–1^, while the peak at *q* ^*^ = 0.04 Å^–1^ persists (). This contrast suggests that the crystalline domains with shorter periodic spacing (*D* ≈ 0.4 nm) are reversibly formed during deformation, while those with longer spacings are not.

The elasticity of the **PHP-x** films was investigated through cyclic tensile testing, involving repeated stretching and releasing of the film up to a maximum strain of 1000% over 10 consecutive cycles (Figure 1e). All samples exhibit a pronounced hysteresis loop in the first cycle, followed by the narrowing of the loop in subsequent cycles. The residual strain of each film increases with each cycle before stabilizing in the range of 200%–570%. Moreover, the stabilized residual strain of **PHP-x** decreases as the value of **x** increases. For example, **PHP-0%** stabilizes at a residual strain of 570%, which decreases to 410% and 260% for **PHP-1%** and **PHP-3%**, respectively, and finally to 200% of **PHP-5%** (Figure 1e). This highlights the significance of more cross-links in improving the elastic recovery of **PHP-x**.

To assess whether the deformation observed during cyclic tensile testing (see Figure 1e, as well as ) leads to permanent damage (i.e., fatigue), the **PHP-x** elastomers were subjected to a thermal relaxation process. Specifically, the previously stretched samples were incubated at 60 °C (above their melting point (*T*_m_)) for 30 min (), followed by a second round of cyclic tensile testing. The resulting cyclic tensile curves were compared with those of the original unstretched samples (see Figures 1f and 1g, as well as ). For **PHP-0%**, **PHP-1%**, and **PHP-3%**, the onset of strain hardening after thermal relaxation occurs at lower strain levels (360%–600%), compared to their respective original values (700%–800%) (see Figure 1f, as well as ). It demonstrates incomplete recovery of the orientated polymer chains, which is also evidenced by the retention of the twisted appearance after the relaxation (). Among them, **PHP-0%** shows the largest difference in the onset point of strain hardening (360% vs 800%) before and after the relaxation at 60 °C (Figure 1f), while differences of 300% and 150% are observed for **PHP-1%** and **PHP-3%**, respectively (). The progressively smaller differences with increasing cross-link density indicate improved chain recovery and structural resilience. Intriguingly, the cyclic tensile curve of the relaxed **PHP-5%** film almost overlaps with that of the initial sample (Figure 1g), demonstrating excellent elastic recovery. This performance highlights the critical role of cross-links in mitigating fatigue and enhancing the durability of **PHP-x** elastomers.

Having confirmed that the cross-linking architecture contributes significantly to enhancing the performance of **PHP-x**, we subsequently evaluated its influence on the material’s depolymerizability. Following the depolymerization processes recently reported by the Sun group,^6,20^ small pieces of **PHP-x** were combined with 2 mol % of G2 (relative to the olefin content) catalyst and 1,3,5-trimethoxybenzene (as NMR internal standard) in CDCl_3_ and then heated at 60 °C for 1 h. The resulting mixture solutions were sent for ^1^H NMR analysis, which allowed us to estimate the **CH** yields. As shown in Figure S14a, all **PHP-x** samples have been successfully depolymerized, generating **CH** again in yields of 77%–92%. Among them, **PHP-0%** achieves the highest yield of 92%, which is comparable to what was reported previously.^20^ Introduction of cross-links leads to a gradual decrease in depolymerization efficiency: **PHP-1%** yields 87%, followed by **PHP-3%** and **PHP-5%** with yields of 78% and 77%, respectively (). This demonstrates that cross-linking indeed weakens the depolymerization capability of **PHP-x**, likely because it leads to the formation of nondepolymerizable bonds. This is indicated in a recent study from the Helms lab, and they revealed that the norbornene part of **DCPD** incorporated in the polymer backbone is not dissociable due to its high ring strain, while the cyclopentene part shows the capability to undergo ring-closing reaction (Figure S15).^45^ Moreover, a new signal at δ = 5.68 ppm appears in the spectra of the depolymerized solutions, which was also noted in the work of Sun et al.^20^ It is attributed to the side product cyclohexene, probably resulting from alkene isomerization occurring on the polymer backbone during depolymerization (). Its yield ranges between 8% and 12% ().

As mentioned above, rubbers are often employed in composite form to enhance their overall performance.^46–48^ Motivated by this, the final part of our study explores the incorporation of a functional filler—activated charcoal (**AC**)—into the **PHP** matrix to create polyolefin-based composites. Specifically, 5 wt % of **AC** was added to the reaction mixture of **CH**, **DCPD** (3 mol %), G2 (0.03 mol %), and **BHT** (1 wt %) prior to ROMP, and it afforded a black composite film termed as **PHP-3%/AC**. The choice of **PHP-3%** as the polymeric substrate is rooted in its highest stress at break among the **PHP-x** series (Figure 1b). DMA traces show that the composite exhibits a similar *T*_m_ to that of the **PHP-3%** parent polymer, but higher *E*′ values across the temperature range measured (Figure 2a), reflecting the improved thermomechanical performance. Tensile testing measurement reveals that **PHP-3%/AC** becomes stronger, and features a higher Young’s modulus of 5.2 MPa as compared to 2.8 MPa of **PHP-3%** (Figure 2b). Subsequent treatments with G2 catalyst and moderate temperature (60 °C) in CDCl_3_ depolymerized the composite networks, while without the catalyst, the composite retained its structural integrity (Figure 2c), suggesting both its chemical resistance and capacity for on-demand depolymerization. ^1^H NMR analysis of the depolymerized solution shows the formation of **CH** in a yield of 60% (Figure 2d).

**Figure 2:**
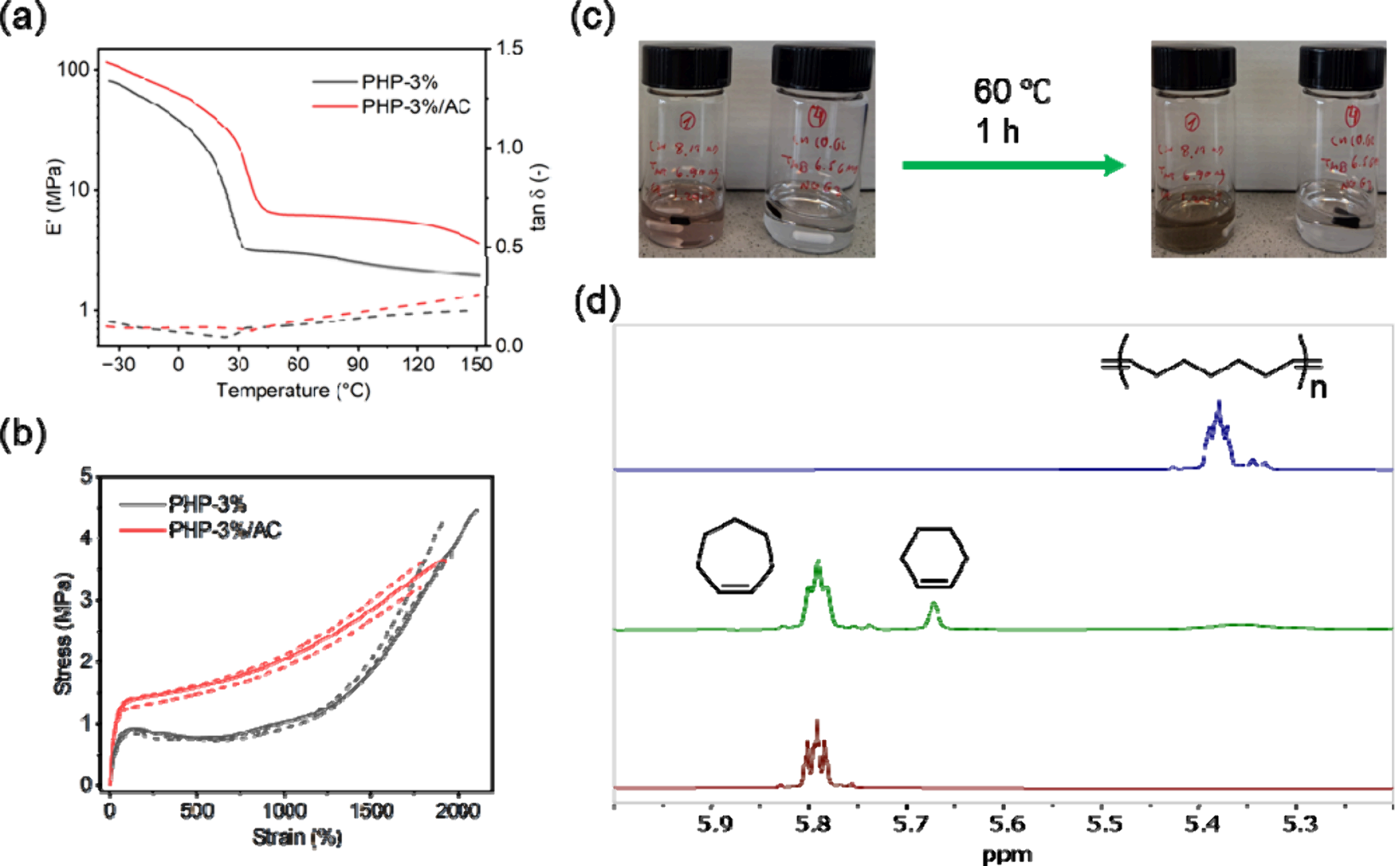
(a) DMA traces showing the storage modulus *E*′ (solid line) and tan δ (dashed line), and (b) stress–strain curves of **PHP-3%** and **PHP-3%/AC** films. (c) Photographs showing a small piece of **PHP-3%/AC** composite dispersed in CDCl_3_ in the presence (left vial) or absence (right vial) of 2 mol % G2 upon thermal treatment at 60 °C for 1 h. (d) ^1^H NMR spectra (400 MHz, CDCl_3_) of starting material **CH** (bottom), depolymerized solution of **PHP-3%/AC** composite (middle), and **PHP-0%** (top).

In conclusion, we have presented a class of superextensible and depolymerizable polyolefins through ring-opening metathesis copolymerization of cycloheptene and dicyclopentadiene. The resulting polyolefins exhibit improved chemical resistance, mechanical strength, thermomechanical properties, and elasticity upon cross-linking. Notably, the polyolefins demonstrate exceptional stretchability, with strain at break reaching up to 2000%. SAXS/WAXS analysis reveals that this superior extensibility arises from strain-induced crystallization within the polymer networks during deformation. While the introduction of cross-linkers and composite fillers improves performance, it compromises depolymerization efficiency. For example, the linear polyolefin exhibits a depolymerization yield of 92%, which decreases first to 78% upon introducing 3 mol % cross-linkers, and further to 60% upon incorporating 5 wt % of activated charcoal. This may catalyze future endeavors to address this performance–depolymerizability tradeoff, for example, through the introduction of reversible cross-linking chemistry. Nonetheless, this work marks a significant step toward the development of high-performance, yet depolymerizable, elastomeric thermosets, potentially contributing to a circular materials economy.

## Supplementary Material





## Notes

Experimental details, materials, instruments, and additional characterization data (PDF)
Supplementary video 1, showing the tensile testing of PHP-3% (MP4)
