# Supplementary material for: Depolymerizable Elastomeric Polyolefin Thermosets with Great Extensibility
Source: ACS Mater Lett. 2025 Dec 8;8(1):145–51. doi: 10.1021/acsmaterialslett.5c01249 (PMC12776579; doi:10.1021/acsmaterialslett.5c01249)
Supplement: Supplementary file 1 [file tz5c01249_si_001.pdf]

Support Information

for

Depolymerizable Elastomeric Polyolefin Thermosets with Great Extensibility

Gadi Slor,[1] Quy Ong Khac,[1] Laura Roset Julià,[1] Youwei Ma,[1*] Francesco Stellacci[1,2*]

[1] Institute of Materials, École Polytechnique Fédérale de Lausanne (EPFL), Lausanne 1015, Switzerland

[2] Institute of Bioengineering, École Polytechnique Fédérale de Lausanne (EPFL) Lausanne 1015, Switzerland

* Corresponding authors: Y. Ma youwei.ma@epfl.ch; F. Stellacci francesco.stellacci@epfl.ch

Materials and Instrumentation

Materials

Cycloheptene (97%, **CH**), dicyclopentadiene (≥96%, **DCPD**), Grubbs Catalyst® 2nd Generation (G2), activated charcoal (**AC,** ~100 mesh particle size), and chloroform-*d* (99.8%, $CDCl_3$) were purchased from Sigma-Aldrich. Dried dichloromethane (99.8%, Extra Dry over Molecular Sieve; DCM) was purchased from ARCOS Organics, and other solvents were purchased from Fisher Chemical. All chemicals were used as received.

Instrumentation

Nuclear magnetic resonance (NMR) spectroscopy. NMR spectroscopy was carried out at 297.2 K on a Bruker Avance DPX 400 spectrometer at frequencies of 400.19 MHz for ^1H nuclei. Spectra were calibrated to the residual solvent peak of $CDCl_3$ (7.26 ppm ^1H NMR). Data were evaluated with the MestReNova software suite (v 12.0) and all chemical shifts (δ) are reported in parts per million (ppm) relative to tetramethylsilane with coupling constant (J) in Hz.

Fourier Transform Infrared (FTIR) Spectroscopy. FTIR was carried out on a Bruker Tensor 27 spectrometer. Samples were analyzed in attenuated total reflectance (ATR) mode using a diamond crystal.

Thermogravimetric analyses (TGA) were performed with a Mettler-Toledo TGA/DSC 1 Stare System. The temperature ranged from 30 °C to 900 °C with a heating rate of 10 °C min^{-1}. Tests were carried out under nitrogen with a flow rate of 40 mL min^{-1}. TGA results were analyzed using the STARe Evaluation software.

Differential scanning calorimetry (DSC) measurements were performed under a nitrogen atmosphere using a Mettler-Toledo STAR system operating at a heating/cooling rate of 10 °C min^{-1} in the temperature range of –80 to 100 °C using a sample mass of ca. 5 mg. The melting temperature, T_m, is reported based on the minimum of the major endothermic melting peak.

Dynamic mechanical analyses (DMA) were performed on the TA Instrument Model Q800 in tension mode. The temperature ranged from –35 to 170 °C with a heating rate of 5 °C min^{-1}, a frequency of 1Hz, and an amplitude of 2% strain.

Tensile testing. Otherwise indicated, stress–strain measurements were carried out at 100 mm·min^{-1} with a Zwick/Roell Z010 tensile tester equipped with a 50 N load cell. Uniaxial tensile tests were carried out with dog–bone-shaped samples with dimensions of 40 × 5 × 0.2 mm (length × width × thickness) that were cut from the solution-casted films according to ASTM D1708. The testing procedure followed the standard method described in ASTM D1708. Cyclic tensile tests were also carried out on the Zwick/Roell Z010 machine with a loading/unloading rate of 100 mm min^{-1}.

Solubility experiment. The weighed samples ($M_{initial}$) were immersed in DCM and shaken in a shaking bed for 24 h. The DCM was changed every 6 h. The solvent was carefully removed with a syringe, and the surface was wiped with a tissue after swelling, after which the samples were weighed ($M_{swelling}$). The samples were dried under vacuum at 60 °C for 24 h to ensure complete removal of the solvent from the material. After drying, the samples were weighed again (M_{drying}). The swelling ratio was defined as $M_{swelling}/M_{initial}$, whereas the gel fraction was defined as $M_{drying}/M_{initial}$.

Small-angle X-ray scattering (SAXS) and wide-angle X-ray scattering (WAXS) analyses were conducted on Xenocs Xeuss 3.0 equipped with a Cu microsource (wavelength Cu-Kα 1.5406 Å) and a Dectris Eiger 1M detector (Xenocs, France). The sample-to-detector distance was 1200 mm for SAXS and 50 mm for WAXS, respectively. The measurements were performed under vacuum, and at room temperature. The exposure time of the samples to the X-ray source was set as 1 hour. Stretched and unstretched films were measured under identical conditions. The 1D $I(q)$ spectra of the film were background subtracted from a spectrum $I(q)$ of the empty holder. No normalization to the film thickness was made. In the measurements coupled with cyclic tensile testing, the sample was first stretched to certain strains before collecting scattering patterns. The exposure time of the samples to the X-ray source was set as 10 min.

Synthesis of polyheptenamer thermosets (PHP-x)

In an argon-filled glovebox, cycloheptene (**CH**), dicyclopentadiene (**DCPD**), and butylated hydroxytoluene (BHT) were weighed in a 20 ml glass vial, with the specific amount for each component shown in Table S1. The mixture was vortexed vigorously, and then 94 μL of 2nd generation Grubbs catalyst (G2) solution in DCM with a concentration of 100 mg mL^{-1} was added. The mixture solution was vortexed vigorously, and transferred to a Teflon mold, and kept at room temperature for 1 h. Then, the mold was taken out of the glovebox and put in a drying oven at 120°C for 30 minutes. After that, a brownish film was formed and termed as **PHP-x**.

The composite was prepared according to the procedure described above, with amounts corresponding to that for synthesizing **PHP-3%**. The only difference is that activated charcoal (194 mg, 5 wt%) was combined with the mixture of **CH**, **DCPD**, and BHT, prior to the addition of G2.

Table S1. Ingredients for the synthesis of **PHP-x**.

Components / Sample name	**CH** [mmol/ g/ mL]	**DCPD** [mmol/ mg/ μL]	G2 [mg/ μL]	BHT [mg]
**PHP-0%**	40 / 3.87/ 4.67	0	9.4/ 94	39
**PHP-1%**	39.6/ 3.81/ 4.62	0.4/ 52.9/ 54.1	9.4/ 94	39
**PHP-3%**	38.8/ 3.73/ 4.53	1.2/ 158.6/ 162.2	9.4/ 94	39
**PHP-5%**	38/ 3.65/ 4.43	2/ 264.4/ 270.3	9.4/ 94	39

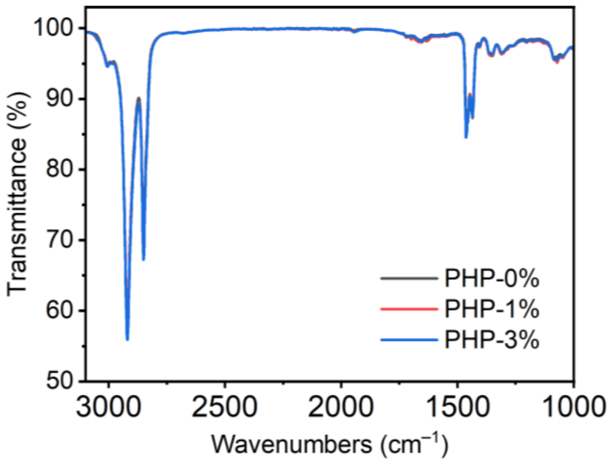

Figure S1. FTIR spectra of **PHP-x**.

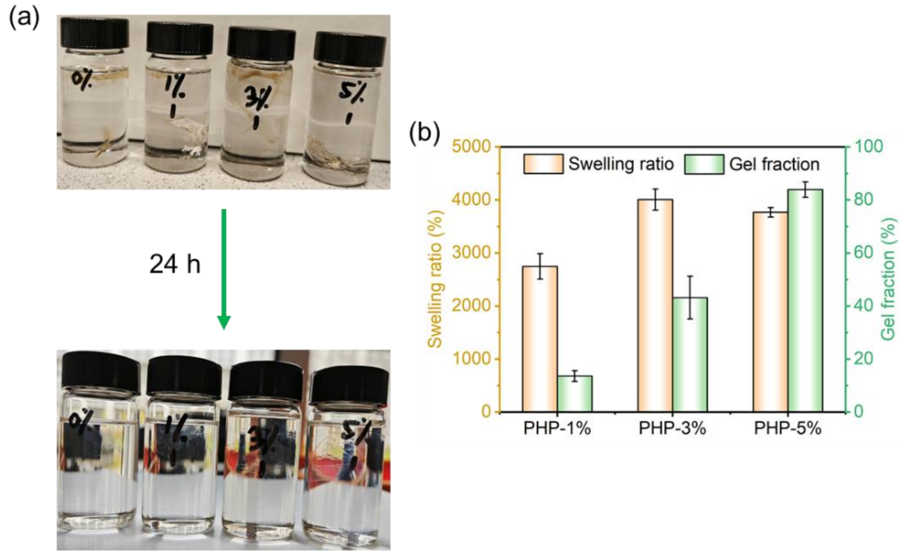

Figure S2. (a) Photographs of **PHP-x** (**x** = 0%, 1%, 3%, and 5% from left to right) dispersed in DCM before (top row) and after (bottom row) a 24h-incubation period. (b) Swelling ratio (yellow) and gel fraction (green) of **PHP-1%/3%/5%**.

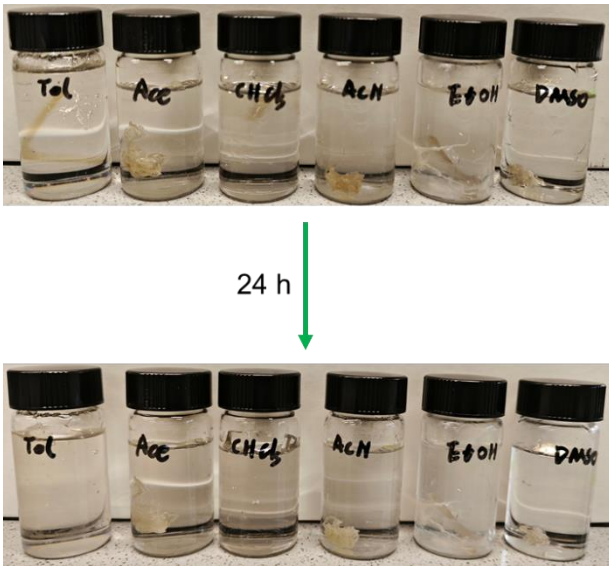

Figure S3. Photographs of **PHP-3%** dispersed in toluene, acetone, chloroform, acetonitrile, ethanol, and DMSO (from left to right) before (top row) and after (bottom row) a 24h-incubation period.

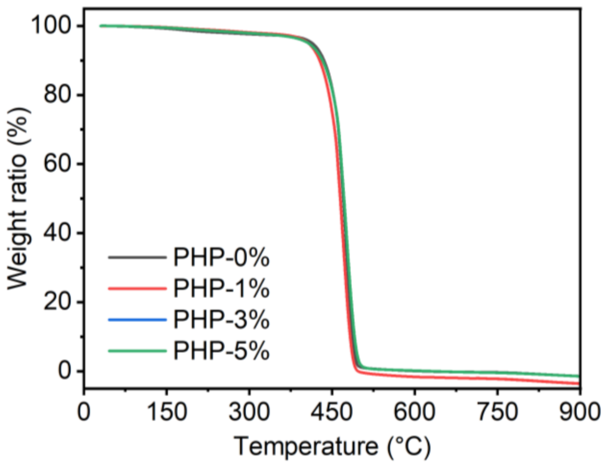

Figure S4. TGA curves of **PHP-x**.

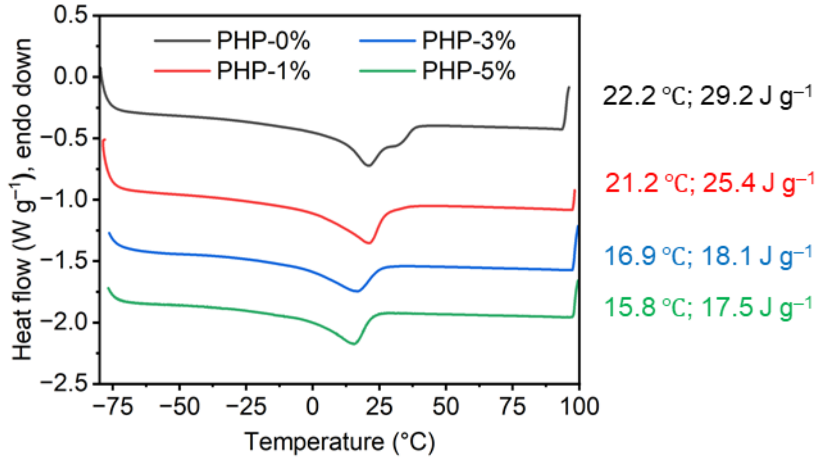

Figure S5. DSC traces of the second heating runs for **PHP-x**, with the melting temperature and fusion enthalpy shown on the right.

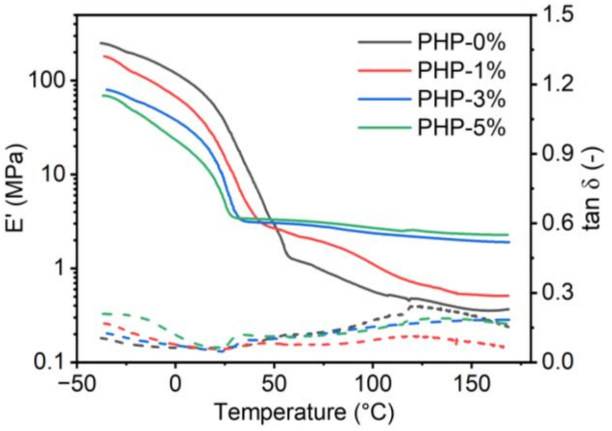

Figure S6. DMA traces showing the storage modulus *E*' (solid line) and tan δ (dashed line) of **PHP-x** films.

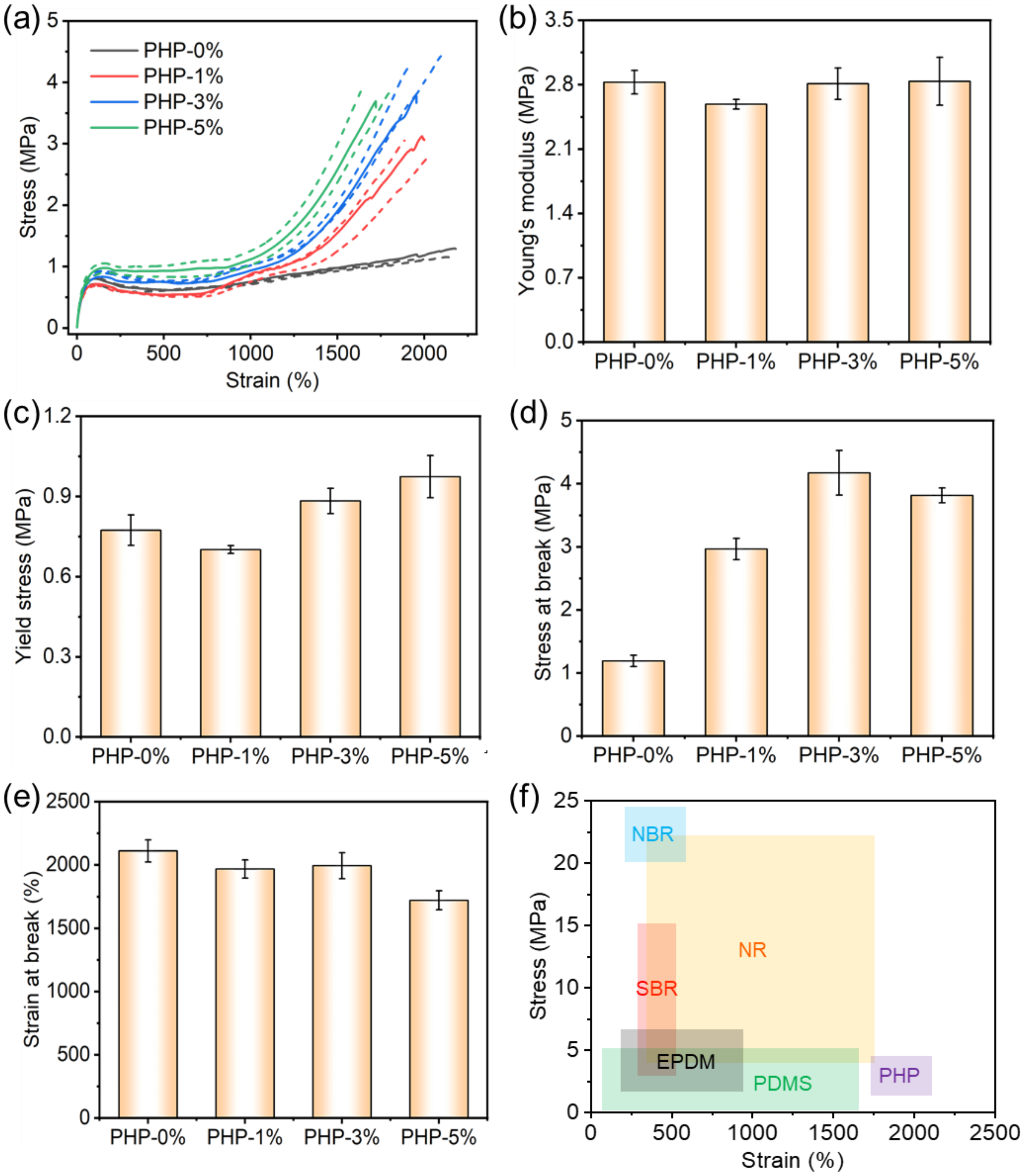

Figure S7. (a) Stress–strain curves, and the associated (b) Young's modulus, (c) yield stress, (d) stress at break, and (e) strain at break of **PHP-x** films. (f) Summary of the stress and strain at break of **PHP** films and several industrially relevant rubbers including natural rubber (NR), styrene-butadiene rubber (SBR), ethylene propylene diene monomer (EPDM), nitrile rubber (NBR), and polydimethylsiloxane (PDMS).

Table S2. Swelling ratio, gel fraction, and mechanical properties of **PHP-x** films.

Sample name	Swelling ratio (%)	Gel fraction (%)	Yield stress (MPa)	Young's modulus (MPa)	Stress at break (MPa)	Strain at break (%)
**PHP-0%**	–	–	0.77 ± 0.06	2.83 ± 0.13	1.19 ± 0.09	2111 ± 87
**PHP-1%**	2746 ± 240	14 ± 2	0.70 ± 0.01	2.59 ± 0.05	2.96 ± 0.17	1968 ± 72
**PHP-3%**	4006 ± 199	43 ± 8	0.88 ± 0.05	2.81 ± 0.17	4.17 ± 0.35	1994 ± 103
**PHP-5%**	3767 ± 91	84 ± 3	0.97 ± 0.08	2.84 ± 0.26	3.81 ± 0.12	1720 ± 75

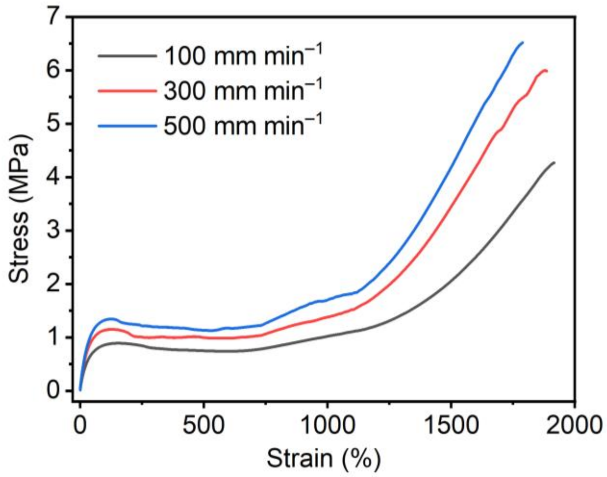

Figure S8. Stress–strain curves of **PHP-3%** films measured at a tensile rate of 100, 300, or 500 mm min^{-1}.

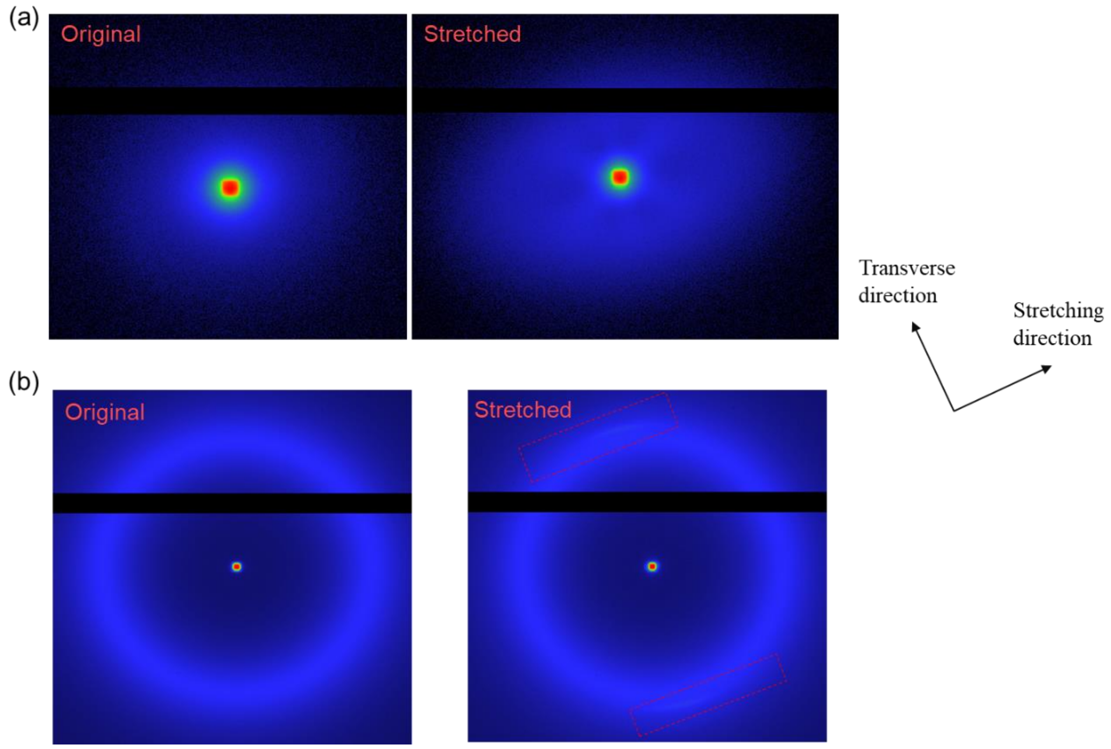

Figure S9. (a) 2D-SAXS and (b) 2D-WAXS patterns of **PHP-3%** films before and after being stretched to breakage, with the stretching direction shown on the right.

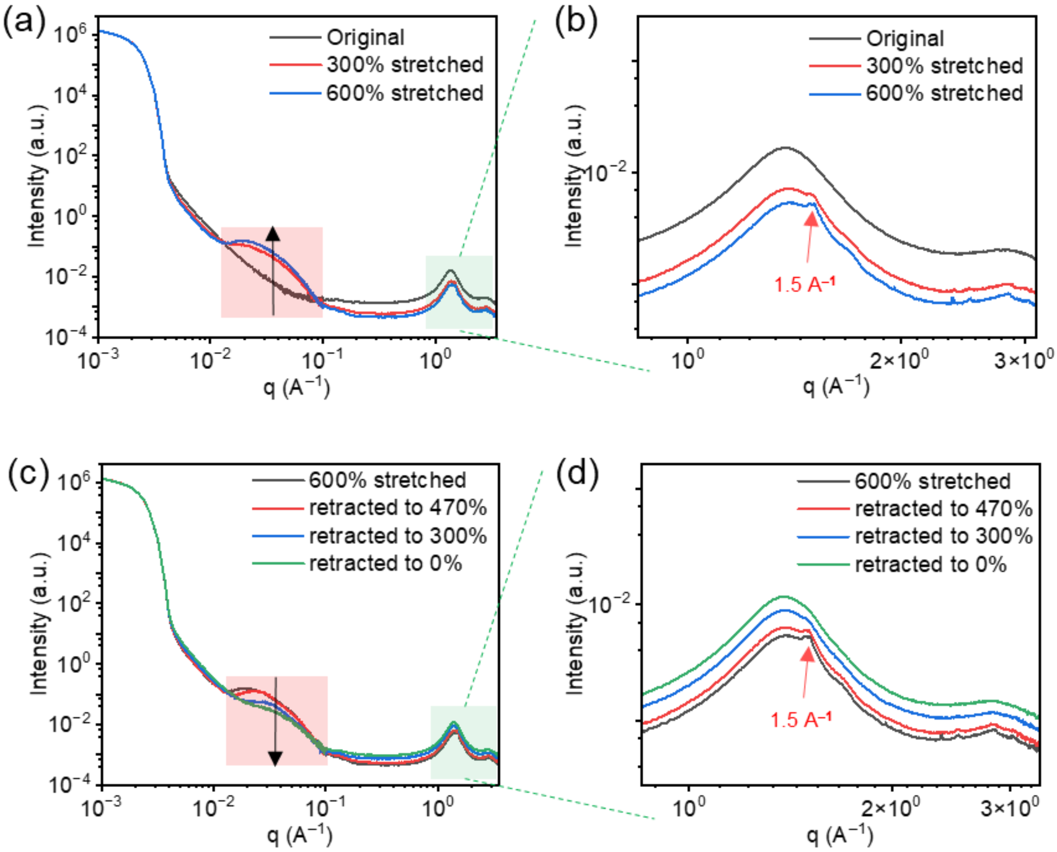

Figure S10. 1D-SAXS/WAXS profiles (a) and their zoom-in (b) of **PHP-3%** film before and after being stretched to a certain strain (as indicated). 1D-SAXS/WAXS profiles (c) and their zoom-in (d) of the stretched **PHP-3%** film before and after being retracted to a certain strain (as indicated).

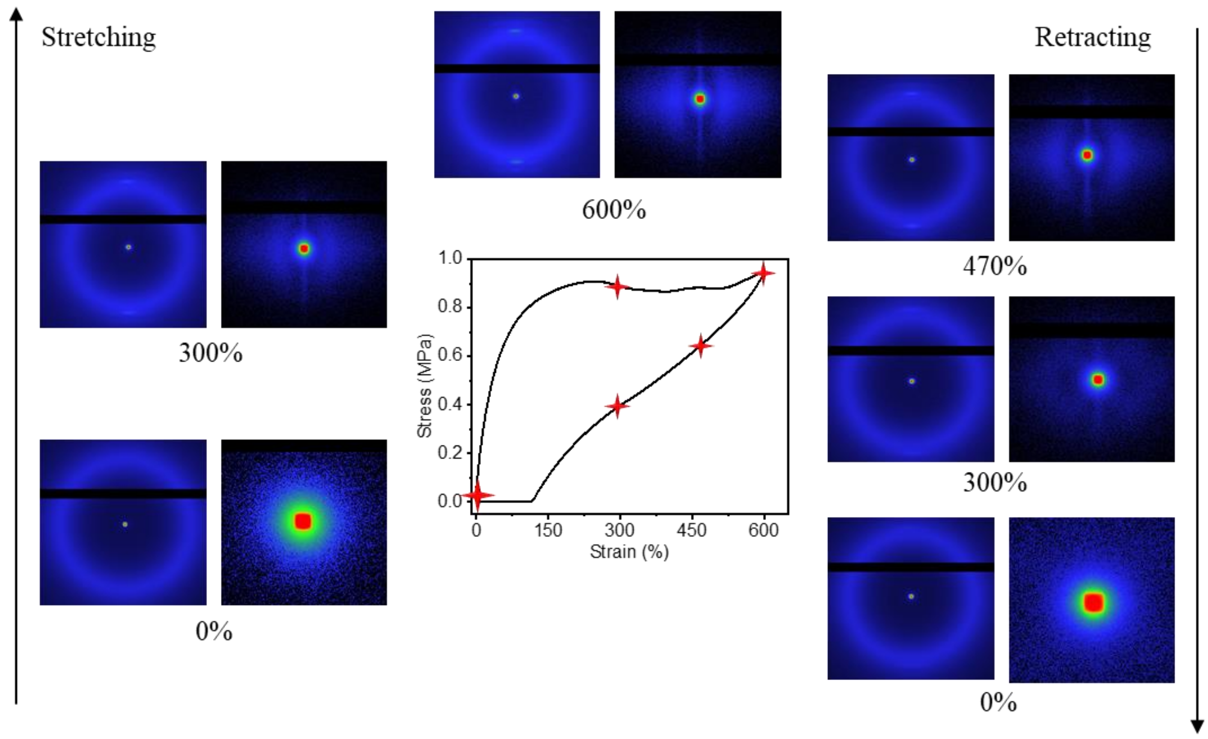

Figure S11. Cyclic tensile curve of **PHP-3%** film at a maximum loading strain of 600% (middle) and its 2D-WAXS/SAXS patterns measured upon stretching the sample or retracting the stretched sample to certain strains (as indicated).

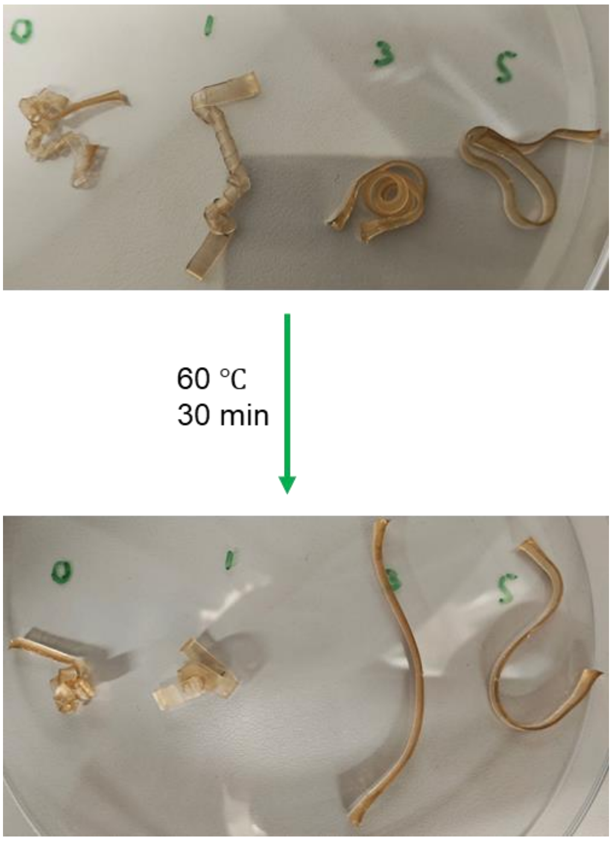

Figure S12. Photographs of **PHP-x** strips (**x** = 0%, 1%, 3%, and 5% from left to right) after undergoing cyclic tensile testing at a maximum loading strain of 1000% for 10 consecutive cycles (top), followed by thermal relaxation at 60°C for 30 min (bottom).

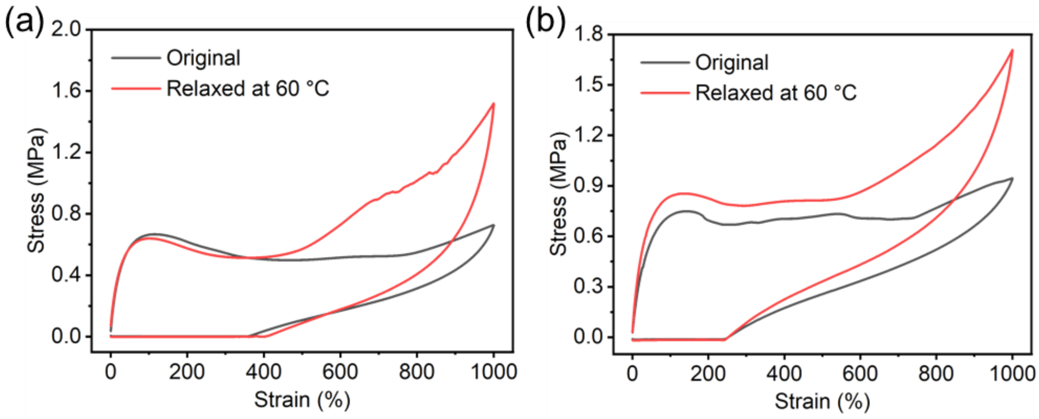

Figure S13. Cyclic tensile curves of the first cycle of (a) **PHP-1%** and (b) **PHP-3%** films before and after undergoing cyclic tensile testing at a maximum loading strain of 1000% for 10 consecutive cycles, and then relaxation at 60 ℃ for 30 min.

Depolymerization of PHP-x and its composite

Thermoset was weighed into a glass vial together with an NMR internal standard, 1,3,5-trimethoxybenzene (TCB), and the vial was taken into an argon-filled glovebox. $CDCl_3$ was added followed by the addition of G2 in 2 mol% to the olefin content. The vial was sealed tightly and taken out of the glovebox. Then, it was heated at 60°C for 1 h while stirring. After that, the sample was cooled to room temperature and sent for ^{1}H NMR analysis. The **CH** yield was calculated using the following equation:

$$m_{CHP} = \frac{I_{CH}}{2} * \frac{m_{TCB}}{168.19\ gr/\ mol} * 96.17\ gr/\ mol$$

Where:

I_{CH} is the integral of the olefinic CH signal,

m_{TCB} is the weight of TCB,

96.17 g mol^{-1} is the molecular weight of **CH**,

168.19 g mol^{-1} is the molecular weight of TCB,

TCB integration was always calibrated as 3.

Then, the depolymerization yield was determined by dividing the **CH** weight in solution by its relative amount in the depolymerization sample.

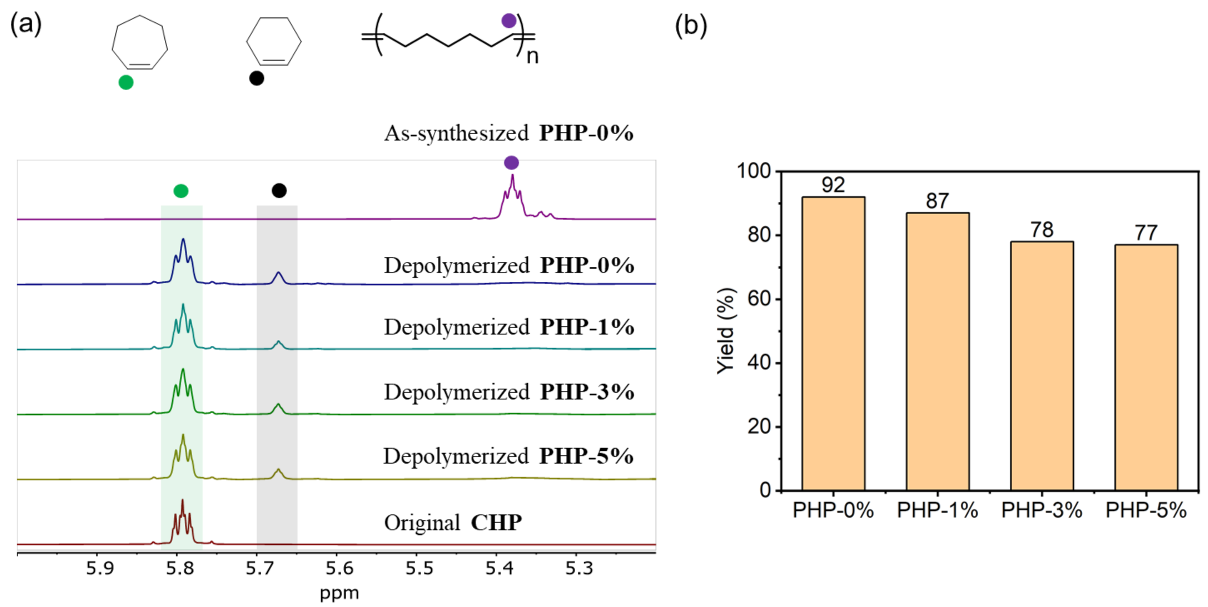

Figure S14. (a) ^{1}H NMR spectra (400 MHz, $CDCl_3$) of starting material **CH**, depolymerized solutions of **PHP-x**, and **PHP-0%** (as indicated). (b) **CH** yield of **PHP-x**.

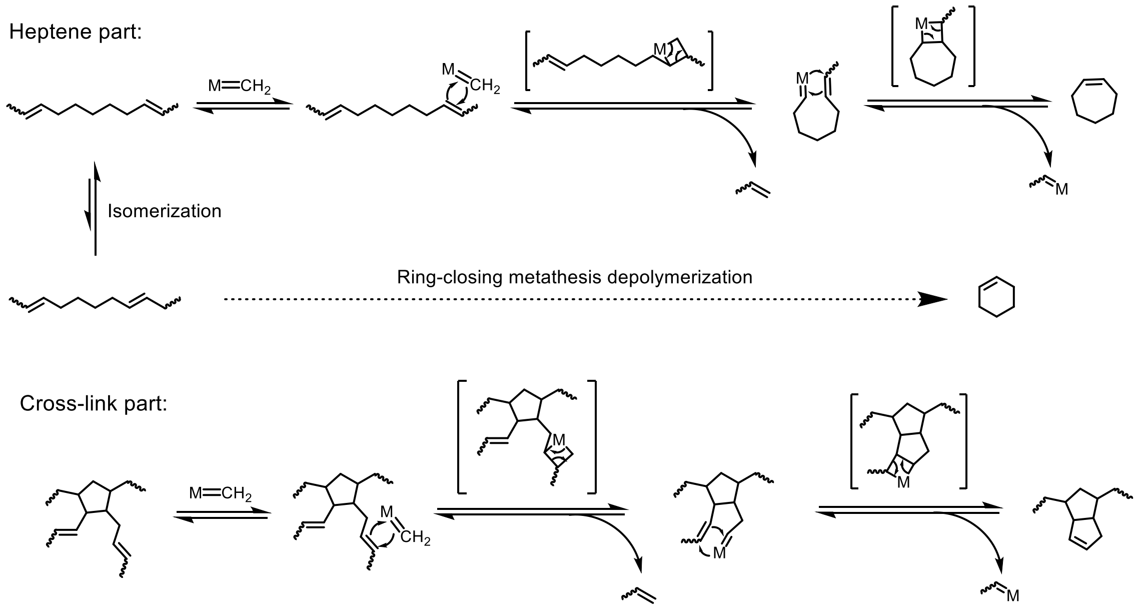

Figure S15. Proposed reaction mechanisms for the ring-closing metathesis depolymerization of the heptene and cross-link parts of **PHP-x**, or the side reactions involving isomerization prior to the depolymerization to form cyclohexene, in the presence of G2 catalyst.
